# Retracted: Intramedullary Chondrosarcoma of Proximal Humerus

**DOI:** 10.1155/2016/1208982

**Published:** 2016-03-24

**Authors:** Case Reports in Radiology

The article titled “Intramedullary Chondrosarcoma of Proximal Humerus” [1] has been retracted as it was found to contain a substantial amount of material, without referencing, from the following published article: “Review: Imaging of Chondrosarcomas,” by L. Ollivier, D. Vanel, and J. Leclère, in Cancer Imaging.
